# Impact of a same day admission project in reducing the preoperative bed occupancy demand in a pediatric inpatient hospital

**DOI:** 10.1016/j.amsu.2022.104304

**Published:** 2022-08-06

**Authors:** Anqaa Almutairi, Hamad Alkhalaf, Angela Caswell, Litaba Efraim Kolobe, Abdulaleem Alatassi, Nezar Alzughaibi, Mohammed Alnamshan, Jubran Alqanatish

**Affiliations:** a Department of Nursing, Ministry of National Guard - Health Affairs, Riyadh, Saudi Arabia; b Department of Pediatrics, Ministry of National Guard - Health Affairs, Riyadh, Saudi Arabia; c Department of Anesthesia, Ministry of National Guard - Health Affairs, Riyadh, Saudi Arabia; d Department of Pediatric Surgery, Ministry of National Guard - Health Affairs, Riyadh, Saudi Arabia; e College of Medicine, King Saud Bin Abdulaziz University for Health Sciences, Riyadh, Saudi Arabia; f King Abdullah International Medical Research Center, Riyadh, Saudi Arabia

**Keywords:** Pediatric inpatient unit, Preoperative bed, Same day admission, BMD, Bed management department, KASCH, King Abdullah Specialized Children's Hospital, PAC, Pre-anesthesia clinic, SDA, Same day admission

## Abstract

**Background:**

A same day admission approach was established for pediatric patients undergoing elective surgery owing to an increase in demand for bed availability and the need for medical, logistical, psychological, and fiscal improvements. This study aimed to assess the effectiveness of the same day admission approach for reducing demand for preoperative bed occupancy in pediatric inpatient units.

**Method:**

Data on elective surgery patients considered for same day admission were prospectively collected in an Excel spreadsheet.

**Results:**

Same day admission patients numbered 269 (25.87%; n = 1040), 461 (41.7%; n = 1104), 382 (38.67%; n = 998), and 560 (44.20%; n = 1267) in 2018, 2019, 2020, and 2021, respectively. Over the 4-year period between 2018 and 2021, pediatric orthopedic surgeries accounted for the majority of same day admissions (29.72%; n = 497), followed by ear, nose, and throat (21.30%; n = 356), general (16.99%; n = 284), plastic (14.53%; n = 243); urology (9.87%; n = 165); optometry and ophthalmology (3.77%; n = 63); neuro (2.51%; n = 42), and dental (1.31%; n = 22) surgeries. The total number of days of saved preoperative beds over the 4-year period was 1672 days (an average of 418 hospital days per year).

**Conclusions:**

This study showed that same day admission approach should be implemented in pediatrics institutions to reduce hospital bed demand. The implementation of this initiative is widely variable between specialties due to interlinked medical, operational, and logistical factors.

**Level of Evidence:**

III.

## Highlights

• Same day admission is a model to reduce the average length of hospital stay for surgical patients and to reduce costs.
• The study is confirming the feasibility of applying the same day admissions' project in the pediatric population.
• This study compares the applicability of the same day admission’s project across pediatrics’ surgical subspecialties.
• This study proves the possibility of applying same day admission's project in the local region.

## 1 Introduction

Bed occupancy refers to the number of hospital beds occupied by patients expressed as a percentage of total beds available in the ward, specialty, hospital, area, or region [[1], [2], [3], [4]]. Bed occupancy rates are among the key performance indicators that are closely followed by hospital stakeholders as a high bed occupancy rate has several implications, such as an increased risk of nosocomial infection, medical errors, and/or operational challenges, particularly during the winter season [[5], [6], [7], [8], [9]].

A plethora of studies have evaluated the risk of high bed occupancy rates on surgery waiting lists and proposed various interventional measures, such as discharge planning, bed management teams, day surgeries, community health services, and same day admission (SDA) of surgical cases [[10], [11], [12], [13], [14], [15]].

This study aimed to determine the benefits of adopting SDA, a process whereby elective surgical patients are admitted to the hospital on the day of their planned surgery [[16], [17], [18]]. SDA (also known as day of surgery admission [ DOSA]) it has been adopted globally and used for decades as a model to reduce the average length of stay and costs associated with surgery [19]. Many developed countries have introduced SDA as a part of their quality improvement projects for pediatric patients [[20], [21], [22]]. In a study by Concannon et al., an SDA project in a single Irish institution for elective surgical inpatients, including the establishment of ring-fenced surgical beds and preoperative assessment clinics in the cost analysis, demonstrated an overall savings of €340,370 [23]. The authors further indicated that since 1977, their institution had used spinal anesthesia for 269 outpatient and inpatient preterm infants who had undergone inguinal hernia repair (as part of the SDA process) to eliminate the need for routine postoperative hospital admission for apnea monitoring [23]. The implementation of this practice reduced the economic expenditure, improved hospital resource utilization, and streamlined surgical service provision. The possibility of reducing the cost of hospitalization has been the rationale behind many similar projects [[24], [25], [26]].

King Abdullah Specialized Children's Hospital (KASCH) is located in Riyadh, Kingdom of Saudi Arabia, and specializes in managing medically complex pediatric cases. The capacity of this hospital is 400 beds, and the current operational number of beds is 220. Of these 220 operational beds, 36 beds are assigned as surgical patient beds. There are 10 operating rooms, and an average of 5000 surgical procedures are performed annually. Out of 5000 cases, approximately 14% (n = 700) are surgical cases admitted as inpatients a day before their surgery for the preparation and completion of the required investigations.

This project was initiated in response to the issues surrounding hospital emergency admissions during the winter season and increased demand for bed availability. Strategies for reducing demand can include the establishment of SDAs to increase bed capacity and improve their utilization [3,8]. Studies have indicated that the length of stay for general surgical patients is influenced by many variables, including direct and indirect medical influences, such as waiting for investigations and planning for home arrangements [[27], [28], [29]].

Although SDA has existed since the 1970s and is used in many Western institutions, to the best of our knowledge, studies evaluating the feasibility of SDA projects in a pediatrics population in Saudi Arabia are lacking. Here, we show the experience of applying SDA in the largest tertiary pediatric hospital in the region to evaluate the positive impact of such an initiative on reducing the demand for preoperative beds in pediatric inpatient units. This project will have a positive impact of reducing the length of stay of surgical cases which, subsequently, will enhance the hospital's operational capacity.

## 2 Methods

### 2.1 SDA initiative

KASCH adopted an SDA initiative as a quality project in 2018 with the primary objective of reducing the demand for preoperative beds and consequently reducing the length of stay of surgical cases. The project was born when a series of meetings were held with hospital leaders such as surgeons, pediatric anesthetists, operation room (OR) service, the chair of bed management, and the surgical nursing team. Together, they formulated the guidelines and outlined the responsibilities of each division for the implementation of SDA. Table 1 shows the map of the SDA process at KASCH.

**Table 1:** King Abdullah Specialized Children's Hospital's same day admission process.

Phase I Preparation	Phase II Day of surgery	Phase III Perioperative and postoperative day
• OR waiting list compiled • Determination of type of surgery by PAC • Surgical coordinators sort inclusive patients • Approval for SDA from bed management	Admission through day surgery care unit to send to OR	Admission to the approved inpatient unit

OR: operation room; PAC: pre-anesthesia clinic; SDA: same day admission.

### 2.2 SDA phases

The implementation of the SDA process included the following three phases. First was the preoperative preparation phase, where patients were first seen by the surgeons in the surgical clinic and placed on the OR waiting list. Then they were referred to the pre-anesthesia clinic (PAC) to be seen by anesthetists for clearance and classification. Following this, the surgical coordinator ensured that the booking process was completed and all the preoperative workups were conducted and completed. All multidisciplinary teams involved in an individual patient's care were consulted before admission orders were obtained through the day surgery unit. Thereafter, every Thursday, the coordinator provided the list to the bed management department (BMD) to approve the proposed list for SDA bed availability.

The second phase was the day of surgery phase, when patients identified in the OR list as SDA cases were admitted through the day surgery unit 2 h before their scheduled surgery time. The SDA patient was prepared for the procedure in the day surgery unit by the nurse. Once the patient was ready, they were sent to the OR for the scheduled procedure.

The third phase was the perioperative and postoperative day phase. Upon the patient's arrival at the post-anesthesia care unit (PACU) from the OR, the BMD was informed within 30 min before discharge to the targeted unit. In the event of a delay, the PACU nurses referred the issue to the BMD supervisor for immediate action. The SDA process ended when the patient was transferred to the targeted unit as an inpatient.

### 2.3 Inclusion and exclusion criteria

A 3-week pilot study was initiated in 2018 to test the reliability of the SDA process. Following the pilot study, the teams were given strict inclusion and exclusion criteria in order to select appropriate patients who were suitable for this process. The patients were included under SDA for orthopedic, pediatrics, plastic, urology, ophthalmology, neuro, dental, and ear, nose, and throat (ENT) surgeries. Furthermore, patients who required: (a) a preoperative medical intervention, (b) a preoperative medical optimization, and (c) an intraoperative massive blood transfusion, especially for scoliosis surgery and craniosynostosis, were excluded.

### 2.4 Ethics and data analysis

Ethical approval for this study was obtained from the King Abdullah Medical Research Centre. A retrospective collection of data related to surgical cases was recorded by the OR statistician in an Excel (Microsoft, Redmond, WA, USA) spreadsheet. Data elements collected were patient demographic information and the type of visit (inpatient or outpatient surgery). Inpatients fell under one of two subcategories: SDA and non-SDA patients. The sample size was calculated based on the inpatient visits for each year. Data were analyzed and illustrated in graphs for each year by specialty. The SDA percentage was calculated by dividing each specialty with the total number of inpatients to reflect the productivity and engagement toward SDA across different surgical subspecialties.

## 3 Results

Table 2 shows the distribution of patients admitted as SDA for the eight pediatric surgical specialties between 2018 and 2021. Of a total of 4399 inpatients, 1672 were admitted as SDA (268, 461, 382, and 560 in 2018, 2019, 2020, and 2021, respectively). The results demonstrate an incremental increase in the percentage of patients admitted as SDA; a slight reduction was observed during the outbreak of COVID-19 in 2020.

**Table 2:** SDAs of all specialties over 4 years (n = 1672).

Year	Distribution of patients between 2018 and 2021					
	SDAs		Non-SDAs		Total patients	
	n =	Percent	n =	Percent	n =	Percent
2018	269	25.87%	771	74.13%	1054	100%
2019	461	41.76%	643	58.24%	1107	100%
2020	382	38.67%	606	61.33%	991	100%
2021	560	44.20%	707	55.80%	1272	100%
Total	1672	38.01%	2727	61.99%	4399	100%

SDAs: same day admissions.

Table 3 shows the distribution of the types of surgery over the entire 4-year study period. Orthopedic surgery accounted for 29.72% (n = 497) of SDAs, followed by ENT (21.3%; n = 356), general (16.99%; n = 284), plastic (14.53%; n = 243), urology (9.87%; n = 165), ophthalmology (3.77%; n = 63), neuro (2.51%; n = 42), and dental surgeries (1.31%; n = 22). During the 4-year period, orthopedics, ENT, and general surgeries were the top three specialties (17–30%) that made optimal use of the SDA project.

**Table 3:** SDAs percentage of each specialty between 2018 and 2021.

Specialty	SDA n =	SDA %
Pediatric Orthopedic	497	29.72
Pediatric ENT	356	21.30
Pediatric Surgery	284	16.99
Pediatric Plastic Surgery	243	14.53
Pediatric Urology	165	9.87
Pediatric Ophthalmology	63	3.77
Pediatric Neurosurgery	42	2.51
Pediatric Dental Surgery	22	1.31
Total	1672	100

ENT: ear, nose, and throat.

## 4 Discussion

The implementation of SDA resulted in the successful reduction of preoperative bed admissions. Hence, over a 4-year study period, 1672 bed days were saved. Certain surgical specialties (i.e., orthopedics, ENT, and general surgery) were consistently able to apply the SDA project in their practice. The study findings show that during the COVID-19 pandemic in 2020, there were more SDA orthopedic patients in comparison to a study done by McIntyre et al. during the pandemic where orthopedics’ SDA surgeries were significantly declined [30]. Further, the higher utilization of operating rooms was not a barrier to apply the practice of SDA since orthopedics was ranked as the specialty with the highest SDA implementation despite performing the second-highest number of surgeries (n = 926), as shown in Table 3. However, certain specialties (e.g., neurosurgery) clearly struggled to apply the SDA practice.

As a general concept, blood group typing and screening results must be available before starting any surgical operation. For the SDA implementation, such blood analysis results had to be available promptly—that was reported as the main challenge during the SDA project. To overcome this challenge, laboratory services agreed to prioritize the SDA blood samples so that the results could be obtained within 70 min of collection. Other minor challenges to the SDA project were related to the (lack of) compliance by some specialties due to the complexity of their patients, but that was managed successfully. Furthermore, the reduced number of postoperative beds that were available during the winter season resulted in extended stays in postoperative recovery rooms, but bed availability was improved through the SDA escalation mechanism.

During the SDA project, due to the unprecedented outbreak of COVID-19, the admission of patients for elective surgeries decreased to such an extent that the full utilization of the SDA project was affected [[31], [32], [33]].

## 5 Limitation and recommendations for further research

The main limitation of this study was that the preliminary cost-saving assessment was conducted based on the initial preoperative period without considering the effect on the overall reduced length of stay of patients admitted under the SDA project. We suggest including patient and family satisfaction surveys in future studies on SDA programs.

## Ethical approval

That is approve by IRB of KAIMRC (King Abdullah International Medical Research Center) with reference number: IRB/2656/21.

Study number: NRC21R/492/11.

## Sources of funding

Non.

## Author contribution

Study conception and design: Anqaa Al Mutairi, Hamad Al Khalaf, Litaba Efraim Kolobe Data acquisition: Anqaa Al Mutairi, Angela Caswell, Litaba Efraim Kolobe, Abdul Aleem Al Atassi, Jubran Al Qanatish. Analysis and data interpretation: Anqaa Al Mutairi, Hamad Al Khalaf, Litaba Efraim Kolobe, Nezar Al Zughaibi, Mohammed Al Namshan Drafting of the manuscript: Anqaa Al Mutairi, Hamad Al Khalaf, Litaba Efraim Kolobe. Critical revision: by all authors: Anqaa Al Mutairi, Hamad Al Khalaf, Angela Caswell, Litaba Efraim Kolobe, Abdul Aleem Al Atassi, Nezar Al Zughaibi, Mohammed Al Namshan, Jubran Al Qanatish.

## Registration of research studies

1. Name of the registry: Impact of a Same Day Admission Project in Reducing the Preoperative Bed Occupancy Demand in a Pediatric Inpatient Hospital
2. Unique Identifying number or registration ID: reviewregistry1407
3. Hyperlink to your specific registration (must be publicly accessible and will be checked): https://www.researchregistry.com/browse-the-registry#registryofsystematicreviewsmeta-analyses/registryofsystematicreviewsmeta-analysesdetails/62d96777773b880023d9a02f/

## Guarantor

Anqaa Almutairi and Dr. Hamad Alkhalaf.

## Consent

Ethical approval for this study was obtained from the King Abdullah Medical Research Centre. A retrospective collection of data related to surgical cases was recorded by the OR statistician in an Excel (Microsoft, Redmond, WA, USA) spreadsheet in security fashion where privacy of information was maintained.

## Previous communication

None.

## Declaration of competing interest

Non.
